# Bearing failure in a mobile bearing unicompartmental knee arthroplasty: an uncommon presentation of an implant-specific complication

**DOI:** 10.1186/s42836-021-00073-9

**Published:** 2021-06-02

**Authors:** Sravya P. Vajapey, Paul M. Alvarez, Douglas Chonko

**Affiliations:** Department of Orthopaedics, The Ohio State University Wexner Medical Center, 241 W. 11th Avenue, Suite 6081, Columbus, OH 43210 USA

**Keywords:** Bearing failure, Mobile bearing, Unicompartmental knee arthroplasty, Bearing dislocation, Bearing fracture, Oxford UKA

## Abstract

**Background:**

We present two cases of unicompartmental knee arthroplasty (UKA) bearing failure in this report—one case of bearing dislocation and one case of bearing fracture. The causes of failure in both cases are evaluated in depth and recommendations are provided regarding intraoperative technique to reduce risk of bearing failure in mobile bearing UKAs.

**Case presentation:**

In the first case, intraoperative evidence of metallosis and chronic pain preceding the traumatic event may indicate that the patient had attenuation of her collateral ligaments that precipitated the instability event. In the second case, the relatively atraumatic nature of the bearing fracture-dislocation and intraoperative evidence of extensive poly wear suggest that the bearing fracture was likely due to a 3-mm bearing selection in the initial surgery.

**Conclusions:**

This case report shows that late bearing in mobile bearing unicompartmental knee arthroplasty can often be a multifactorial event and treatment must address all the risk factors that led to bearing dislocation. Bearing fracture is a very rare complication associated with mobile bearing UKA and patients with thin polyethylene inserts are at risk for bearing fracture even in the absence of poly wear.

## Background

Unicompartmental knee arthroplasty (UKA) is a successful procedure that has been in use since 1982 [1]. Studies have shown that modern UKA has a survival rate of greater than 90% at 10 years [2, 3]. UKA has been divided into two primary types: the first type utilizes a fixed bearing polyethylene insert, and a second type utilizes a mobile bearing polyethylene insert. While there are no differences in long-term outcomes or overall complication rate between the two types of UKA, there are design differences between the two implants that lead to different types of complications with each type of implant [4]. In the mobile bearing (Oxford) UKA, the polyethylene insert functionally mimics the native meniscus and is fully congruent throughout the range of motion of the knee [5]. While the mobile bearing UKA confers the biomechanical advantage of achieving near-normal knee kinematics, it also comes with a risk of dislocation or fracture of the meniscal bearing—a complication that is unique to this implant design [6].

Aseptic loosening is the most common complication in fixed bearing UKAs while bearing dislocation is the most common complication after mobile bearing UKA [7]. Bearing dislocation has been reported to be the cause of failure in 5–11% of mobile-bearing UKAs requiring revision surgery [8, 9]. Possible causes of bearing dislocation or failure include malposition of components, flexion-extension gap mismatch, instability due to cruciate or collateral ligament attenuation, poly wear due to many years of use, excess mobility due to thinner bearing selection in surgery, or impingement of the polyethylene insert against bone or implant [10].

We present below two cases of late meniscal bearing failure. The first case involves a traumatic bearing dislocation and the second involves an atraumatic bearing fracture/dislocation following a medial mobile bearing (Oxford) UKA. The causes of failure in both cases are evaluated in depth and recommendations are provided regarding intraoperative technique to reduce risk of bearing failure in mobile bearing UKAs.

The patients whose cases are presented below were informed that data concerning the case would be submitted for publication, and they provided informed consent.

## Case presentation 1

### History and examination

**Fig. 1:**
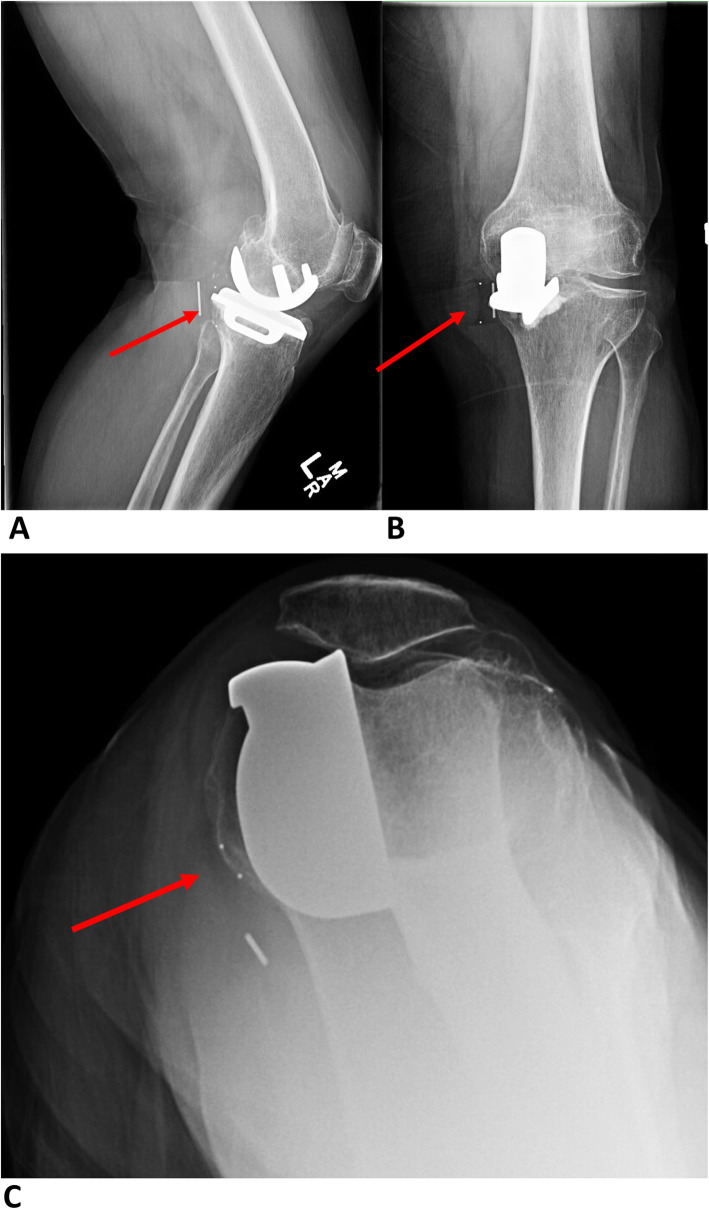
(**a**) Lateral, (**b**) AP, and (**c**) sunrise radiographs of the left knee demonstrating a cemented medial Oxford unicompartmental knee arthroplasty with posteromedial dislocation of the polyethylene mobile bearing insert. There is also significant lateral and patellofemoral osteoarthritis with associated anterior femoral and patellar osteophytosis

A 76-year-old woman (BMI = 21.01 kg/m^2^) was referred to our Joint Arthroplasty clinic by her primary care physician (PCP) due to acute left knee pain and stiffness. She said her left knee felt unstable and buckled, resulting in a ground level fall. Since then, she had pain with weight bearing and decreased range of motion. Prior to her fall, she was ambulatory without assistance but experienced intermittent pain and swelling in her left knee for several months. She did relate that she had experienced multiple episodes of instability on stairs and uneven surfaces and several near-falls due to her left knee. She had a past medical history significant for left knee arthritis and underwent a left medial mobile bearing UKA 5 years prior to presentation. On examation, she had a mild left knee effusion with no tenderness to palpation. Her left knee ROM was 30 to 120 degrees with significant instability with varus and valgus stress at 300 and 900. She had a varus deformity and was neurovascularly intact. Radiographs of the left knee demonstrated a cemented medial Oxford unicompartmental knee arthroplasty with well-fixed femoral and tibial components and a posteromedial dislocation of the polyethylene insert (Fig. 1).

After a thorough review of her history, imaging, and examination records, an extensive discussion was held with the patient regarding various operative treatment options. Given her development of both lateral and patellofemoral arthritis, attenuation of her collateral ligaments and failure of her unicompartmental knee arthroplasty, revision surgery with left total knee arthroplasty (TKA) was recommended. The risks of surgery were discussed, and included, but were not limited to: iatrogenic fracture, knee stiffness, bleeding, infection, need for further surgery, further attenuation of the collateral ligaments and recurrence of pain. She understood the risks and gave informed consent to proceed with revision surgery.

### Intraoperative details

The patient underwent general anesthesia. A standard medial parapatellar approach was used to expose the knee joint. Significant metallosis was appreciated within the synovium and a complete synovectomy was performed. The polyethylene insert was found in the posterior-medial compartment of the knee and subsequently extracted. The Oxford unicompartmental arthroplasty components were removed by interrupting the cement-prosthetic interface with both a reciprocating saw and flexible osteotomes. There was minimal bone loss with component removal; however significant stress shielding and osteopenia were found behind both the femoral and tibial components. The Zimmer (Warsaw, IN, USA) Biomet Vanguard 360 Revision Knee System was used. A 63-mm constrained posterior stabilized tibial component with a 14 × 40 mm tibial stem and a 60-mm femoral component with a 18 × 40 mm femoral stem were inserted. Ten-mm tibial augments and a 5-mm distal medial femoral augment were used to achieve stability in flexion and extension. A 12-mm polyethylene insert was used to balance the flexion and extension gaps. The patella was resurfaced. Good mid-flexion stability and excellent patellar tracking were noted intraoperatively. The wound was then closed in a layered fashion, and the patient was awoken from anesthesia without complications.

### Postoperative course

**Fig. 2:**
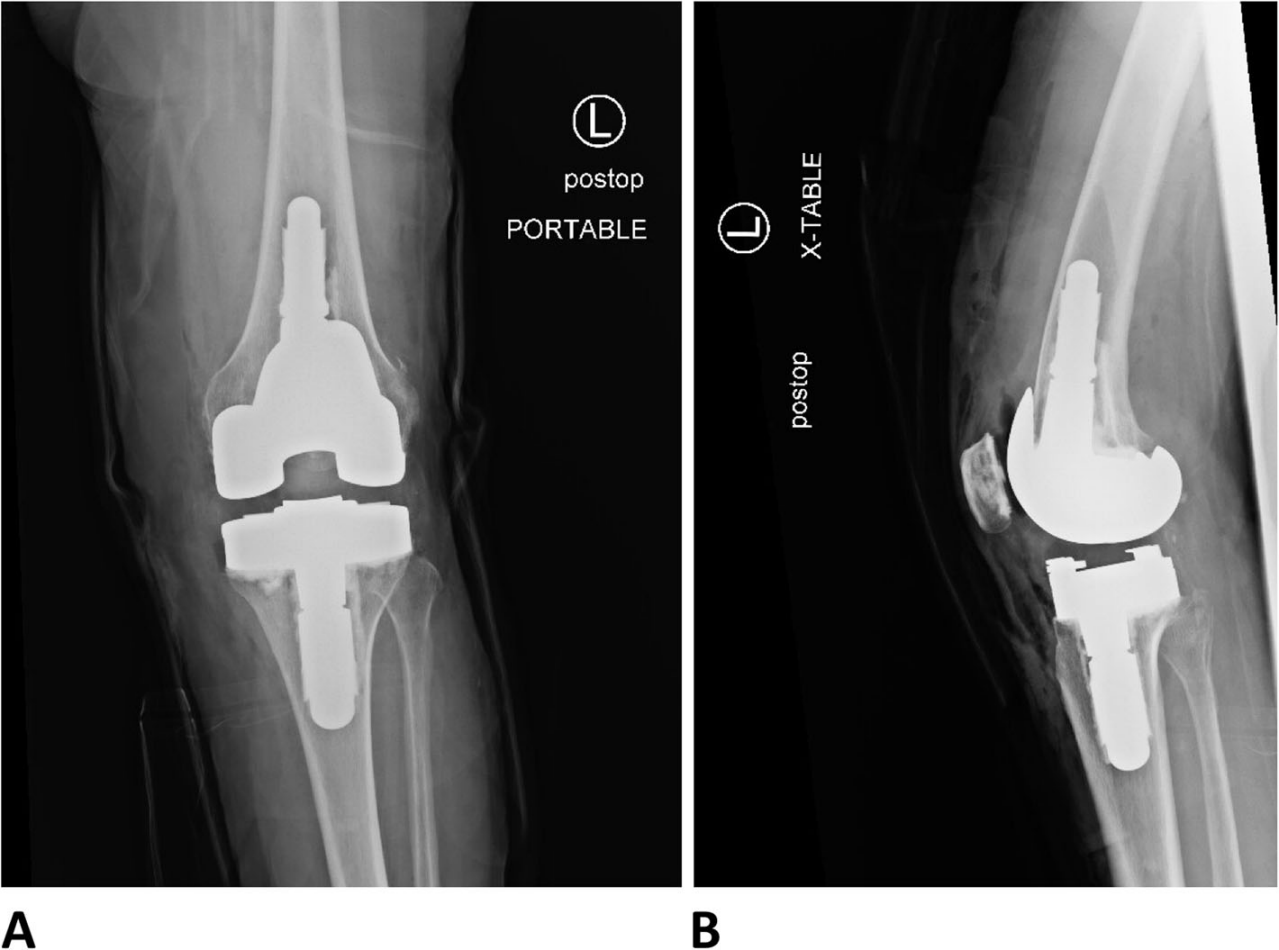
Postoperative radiographs demonstrating successful revision to a total knee arthroplasty using the Zimmer Biomet Vanguard 360 Revision Knee System (**a**) AP view (**b**) lateral view

Imaging of the left knee obtained postoperatively showed appropriate alignment of TKA implants (Fig. 2). The patient received 24 h of intravenous (IV) antibiotics and appropriate venous thromboembolism (VTE) prophylaxis postoperatively. She was allowed to weight-bear as tolerated. She received physical therapy while inpatient and after discharge to home on postoperative day (POD) 0. At her most recent clinic visit 2 weeks after surgery, she was participating in home physical therapy, ambulating well with the assistance of a cane, and her pain was well controlled. She was neurovascularly intact and the incision was healing well.

## Case presentation 2

### History and examation

**Fig. 3:**
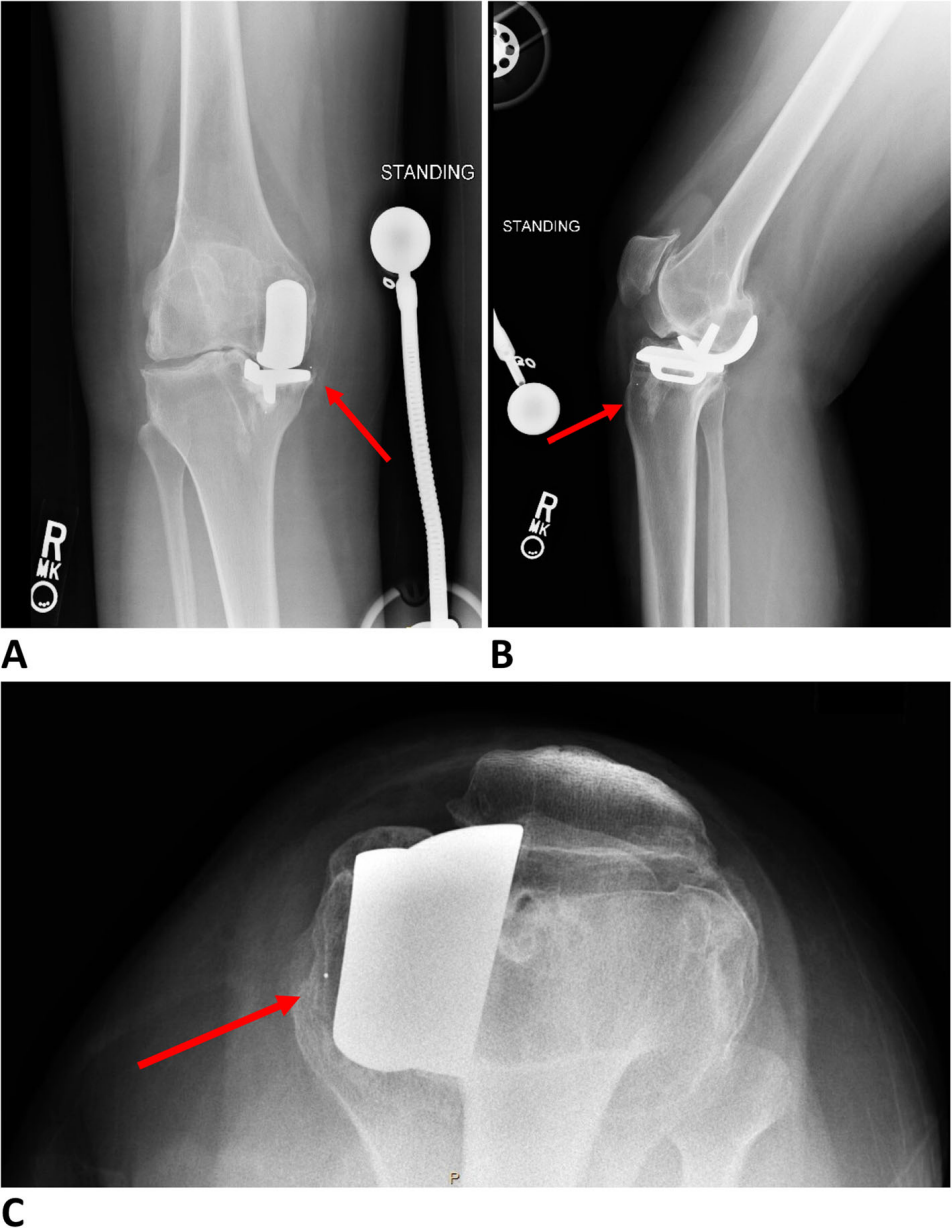
(**a**) AP view (**b**) lateral view (**c**) sunrise views of the right knee demonstrating a cemented right medial Oxford UKA with a broken and medially dislocated polyethylene insert. Significant lateral and patellofemoral osteoarthritis with evidence of anterior femoral osteophytes can also be appreciated

A 40-year-old woman (BMI = 32.28 kg/m^2^) presented to our joint arthroplasty clinic with progressive atraumatic right knee pain and effusions lasting over several years. She had a history of right knee medial mobile bearing UKA performed 10 years prior to presentation. She complained of acute worsening of her chronic right knee pain and decreased ROM. She endorsed experiencing popping sensation while ambulating. On examation, her right knee was tender to palpation over the medial joint line. Her ROM was 0 to 110 degrees with instability appreciated with manual varus and valgus stress at full extension and mid-flexion. She had no obvious deformity and was neurovascularly intact. Radiographs of the right knee demonstrated a cemented medial Oxford unicompartmental knee arthroplasty with a broken and dislocated polyethylene insert. Significant lateral and patellofemoral osteoarthritis with evidence of anterior femoral and patellar osteophytosis was appreciated (Fig. 3).

After a thorough review of her history, imaging, and examation records, an extensive discussion was held with the patient regarding various operative treatment options. Given her development of both lateral and patellofemoral arthritis and failure of her UKA bearing, we recommended a revision to a right TKA. The risks of surgery of surgery were discussed, and included, but were not limited to: iatrogenic fracture, knee stiffness, bleeding, infection, need for further surgery, further attenuation of the collateral ligaments and recurrence of pain. She understood the risks and gave informed consent to proceed with revision surgery.

### Intraoperative details

**Fig. 4:**
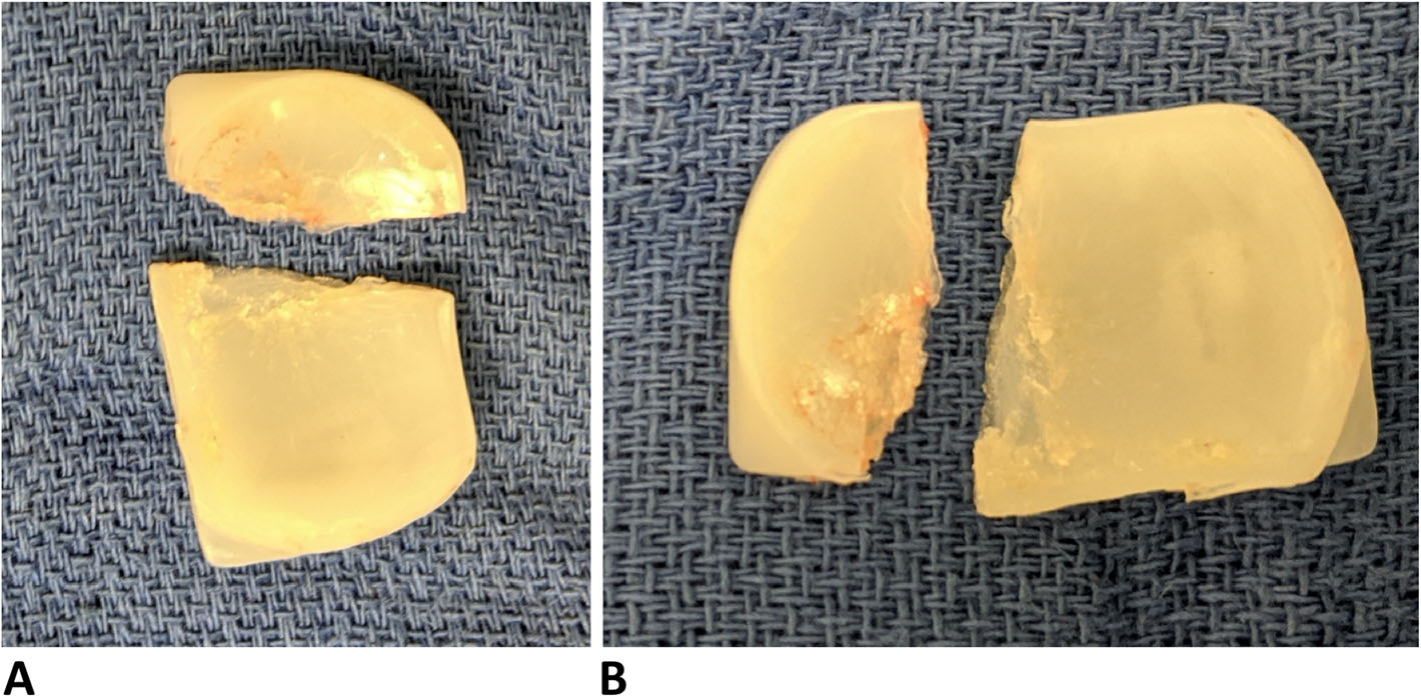
Broken polyethylene insert with significant anterior and central wear

The patient underwent general anesthesia, and a standard medial parapatellar approach was used to expose the knee joint. Significant synovitis along with poly wear debris was noted, and a complete synovectomy was performed. The polyethylene insert was noted to be broken and dislocated, with pieces lodged in the medial and posterior knee. The polyethylene insert used was 3 mm, the thinnest size available. There was significant central wear at the site of poly insert fracture (Fig. 4).

Once the broken pieces of the polyethylene insert were removed, the Oxford UKA femoral and tibial components were removed by interrupting the cement-prosthetic interface with both a reciprocating saw and ¼ inch osteotomes. There was minimal bone loss with the removal of the hardware. The Zimmer Biomet Vanguard Complete Knee System was used and a 71-mm cruciate retaining tibial component and a 65-mm femoral component were inserted. Two screws were placed at the keel site of the previous Oxford tibial plate to act as rebar for the bone cement secondary to the patient’s osteopenia.

A 16-mm polyethylene insert was inserted to balance the flexion and extension gaps with adequate mid flexion stability. The patella was resurfaced. The wound was then closed in a layered fashion, and the patient was awoken from anesthesia without complications.

### Postoperative course

**Fig. 5:**
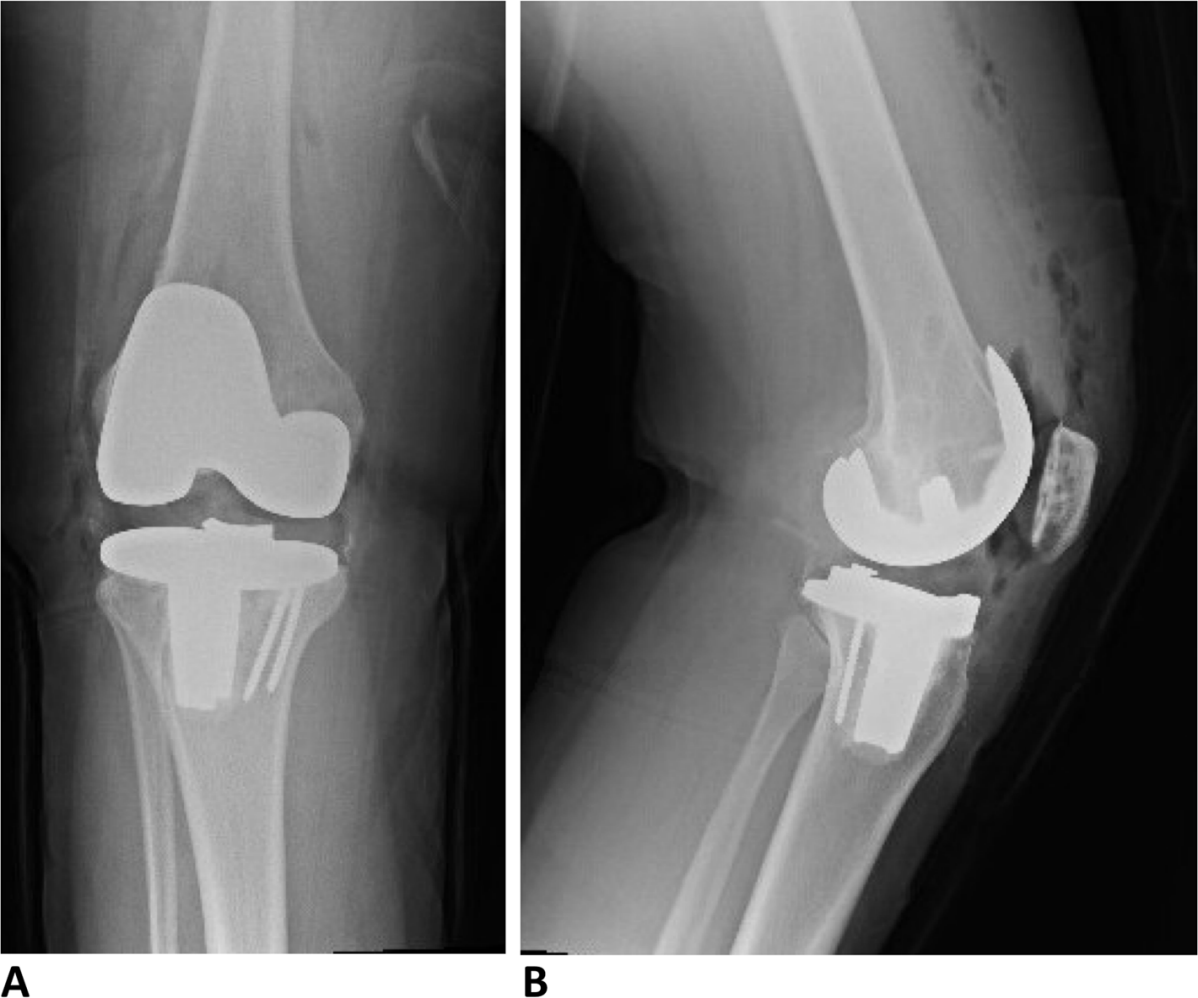
Postoperative radiographs demonstrating appropriate alignment and position of total knee arthroplasty implants on (**a**) AP view and (**b**) lateral view

Imaging of the right knee was obtained postoperatively and showed appropriate alignment and position of the implants (Fig. 5). The patient received 24 h of intravenous (IV) antibiotics and appropriate VTE prophylaxis postoperatively. She was allowed to weight-bear as tolerated immediately after operation. She received physical therapy while inpatient and after discharge to home on POD1. She was doing well at her last clinic visit 2 weeks after surgery with well healed incision.

## Discussion

While aseptic loosening and osteoarthritis progression are the most common causes of failure after UKA, bearing failure in the form of dislocation or fracture is not uncommon and occurs in about 11% of patients [8, 9]. Bearing dislocation, which is more commonly reported in mobile-bearing UKA, can occur due to a multitude of causes, including residual knee deformity, collateral ligament insufficiency, component malpositioning, impingement, repetitive deep knee flexion or infection [10]. Regardless of whether the bearing dislocation was traumatic or atraumatic, patients who experience this complication after UKA often have predisposing conditions that are multifactorial in nature. For instance, Kawaguchi et al. reported a case of meniscal bearing dislocation in a patient who rolled over while sleeping [11]. The cause of failure in their case report was hypothesized to be a combination of small femur, small tibial component, and valgus knee deformity. Apostolopoulos et al. reported a case of atraumatic bearing dislocation 5 years after UKA [12]. The cause of failure in their report was hypothesized to be aseptic loosening leading to metallosis and bearing instability. Similarly, in our case report, we described a patient who sustained an atraumatic bearing dislocation after months of chronic instability. The intraoperative evidence of metallosis and the patient’s chronic pain preceding the dislocation event suggest that the patient suffered from an attenuation of collateral ligaments that led to chronic instability and wear that precipitated the bearing dislocation.

This multifactorial nature of a bearing dislocation in UKA is important to recognize because revision surgery can result in a high failure rate and re-dislocation if all the predisposing factors for bearing dislocation are not addressed at the time of revision surgery. For instance, Kim et al. reported re-dislocation in 21% of the patients treated with a simple bearing exchange in their retrospective study of 1576 patients who underwent UKA [7, 13]. This high rate of dislocation was hypothesized to be due to failure to address the initial cause of bearing dislocation. Therefore, as a general rule, revision to TKA is recommended in patients with incompetent collateral ligaments, significant coronal or sagittal plane deformity, progression of arthritis in the other compartments, or chronic prosthetic joint infection [14]. However, bearing exchange is a reasonable option where the underlying cause of dislocation is solely overstuffed medial compartment (treated by downsizing the bearing), flexion instability (treated by upsizing the bearing), or impingement due to osteophytes (treated by removing excess bone or osteophytes) [15].

While bearing dislocation in mobile bearing UKA is not an uncommon complication, bearing fracture has only ever been reported twice in the literature so far [16–18]. The causes of bearing fracture in mobile bearing UKA include increased poly wear, use of thin poly in the initial surgery, and use of earlier design of the Oxford knee, which had higher degrees of freedom and instability of polyethylene insert [10, 19]. Lim et al. reported a case of bearing fracture in a patient 7 years after UKA with a phase III Oxford implant [20]. The mode of failure in their case was uneven delamination of the polyethylene in the thinnest articular portion of the insert, leading to a fatigue crack that propagated [19]. Munjal et al. reported bearing fracture in an obese patient 7 years after a phase III Oxford UKA due to sudden excessive load on a thin insert (3 mm) weakened by oxidation [1]. Both of these cases are similar to the second case presented in our case report in that all three cases occurred late and the thinnest polyethylene insert was used in the initial surgery. Zimmer Biomet changed the shape of the polyethylene insert in their later phases of design primarily in the anterolateral portion to make it more difficult to spin and dislocate [21]. The original phase 1 design of the Oxford partial knee had more degrees of freedom and instability of the polyethylene insert. This may have led to increased wear and delamination and subsequently to catastrophic failure in these earlier designs [22]. Our case report and prior published studies suggest that use of a thin polyethylene insert (less than 4 mm in size) places the patient at increased risk of bearing fracture, especially with use of earlier designs of the Oxford knee.

Treatment options for catastrophic bearing failure due to fracture include bearing exchange and TKA. Revision to TKA should be considered if the bearing failure occurred due to increased contact stresses secondary to varus alignment of the knee, component malalignment, or morbid obesity of the patient [23]. Bearing exchange can be considered if the underlying cause of failure was solely too thin a polyethylene liner. However, outcomes after bearing exchange for polyethylene wear are not well studied and long-term survivorship is unknown.

## Conclusion

Bearing dislocation and bearing fracture in mobile-bearing UKA represent uncommon causes of failure. This case report and prior studies show that late bearing dislocation in mobile bearing UKA is often a multifactorial event, and bearing exchange alone can result in high re-dislocation rate if all predisposing factors that lead to bearing dislocation are not addressed at the time of revision surgery. As described in our case report, revision to TKA remains an appropriate treatment option with excellent long-term outcomes in most cases of bearing dislocation. Bearing fracture is a rare complication that has been infrequently described in the literature. Patients with a thin polyethylene insert are at higher risk for bearing fracture even in the absence of poly wear, especially with earlier designs of the Oxford UKA. Treatment for bearing fractures includes bearing exchange and revision to TKA.

## Data Availability

All data generated or analyzed during this study are included in this published article and its supplementary information files.

## Abbreviations

PCP: Primary care physician
POD: Postoperative day
ROM: Range of motion
TKA: Total knee arthroplasty
UKA: Unicompartmental knee arthroplasty
VTE: Venous thromboembolism
